# Parasitological transitions: selected outcomes from the XXXII Congress of the Italian Society for Parasitology

**DOI:** 10.1017/S003118202300104X

**Published:** 2023-10

**Authors:** Rudi Cassini, Fabrizio Bruschi, Antonio Frangipane di Regalbono, Laura Rinaldi

**Affiliations:** 1 Department of Animal Medicine, Production and Health, University of Padova, Viale dell'Università, 16 – 35020 Legnaro, Italy; 2 Department of Translational Research, N.T.M.S., School of Medicine, University of Pisa, Pisa, Italy; 3 Department of Veterinary Medicine and Animal Production, University of Naples Federico II, 80137 Naples, Italy

**Keywords:** congress, Italy, medical parasitology, society, veterinary parasitology

## Abstract

Founded in 1959, the Italian Society of Parasitology (SoIPa) includes nearly 200 researchers and professionals in the fields of medicine, veterinary medicine, biotechnology, epidemiology and environmental sciences. The diversity of its members, in a historical and continuous collaboration with other international scientific societies, embodies a broad and multidisciplinary field such as parasitology. Since 1959, SoIPa has organized a biennial congress, covering all aspects of general parasitology with participants from all over Italy, Europe and beyond, involved in a dynamic and multi-faceted scientific framework of contributions and symposia. The present Special Issue (SI) contains 6 review papers and 1 research article, focussed on emerging topics presented and discussed during some of the symposia organized within the XXXII SoIPa Congress, held in Naples from 27th June to 30th June 2022. These review papers reflect several emerging subjects (i.e. ‘Italian network on Neglected Tropical Diseases’, ‘Wildlife parasites and citizen science’, ‘Comparing approaches to parasitological issues’, ‘Unusual perspectives on the role of parasites’) with the aim to explore the new role that parasitologists can play in the future society, working together to promote dialogue on science-informed decisions to support the so-called ‘twin green and digital transition’.

## Graphical abstract

**Figure:**
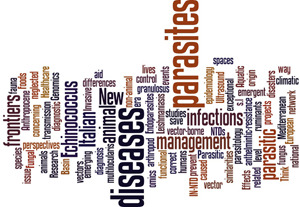

## Introduction

Parasitology is a broad and cross-cutting discipline, whose multidisciplinary nature has already been recognized by several scientists and by several national and international scientific societies (Blake and Betson, 2017; Stothard *et al*., 2018). The Italian Society for Parasitology (***So****cietà **I**taliana di **Pa**rassitologia*, hereafter ‘SoIPa’) embodies this multidisciplinarity through the diversity of its members, which include scientists with medical, veterinary, biotechnology, epidemiology and environmental science backgrounds. *‘Italy has the longest history in modern parasitology, based on the researches, first of Francesco Redi, the begetter of chemotherapy, Cestoni and Spallanzani, of Lancisi and Bassi and of countless others*’ (Garnham, 1971)…this is the incipit of the review article by Roncalli Amici (2001), which describes in detail the history of Italian parasitology from Roman times to the 21st century.

SoIPa (www.soipa.it) was founded in 1959 in continuity with the Society for the Study of Malaria (established in 1898).The first president was Ettore Biocca (1959–1975; second term 1982–1984), followed by other eminent professors of parasitology and parasitic diseases, including Paolo Magaudda (1976–1978), Bruno Baldelli (1979–1981), Silvio Pampiglione (1985–1988), Mario Coluzzi (1989–2000), Claudio Genchi (2001–2008), Mario Pietrobelli (2009–2016) and Fabrizio Bruschi (2017–2020, 2020–present).

The first Congress of SoIPa was held in Sassari (Sardinia Island) in September 1959. In the same year, the journal ‘Parassitologia’ was founded as the official journal of the Society. Unfortunately, after 52 volumes, this journal ceased publication in 2010, despite its reputation at an international level.

SoIPa's mission is to strengthen scientific collaboration, develop joint research agendas, coordinate research on parasites of medical and veterinary importance and promote parasitological research in Italy, Europe and beyond. SoIPa also promotes and supports the teaching of parasitology throughout Italy. SoIPa's goals are pursued through the integration of ideas, skills and expertise among members (~200) and collaboration with other international scientific societies. SoIPa aims to advance and promote networks for standardization of diagnostic parasitological methods and harmonization of surveillance approaches and control strategies for parasitic infections.

SoIPa is affiliated to the World Federation of Parasitologists (WFP), the European Federation of Parasitologists (EFP), the Italian Society of Veterinary Sciences (SISVet) and the Federation of Italian Medical-Scientific Societies (FISM). SoIPa is also closely connected with the World Association for the Advancement of Veterinary Parasitology (WAAVP) and has recently endorsed the World Health Organization's initiative ‘Ending NTDs: together towards 2030’, signing the Kigali declaration (https://unitingtocombatntds.org/en/the-kigali-declaration/). Other SoIPa activities include continuing education courses and thematic symposia and workshops. In addition, the Society sponsors awards for early career researchers in the field of medical and veterinary parasitology and promotes the ‘Spring Parasitology’ series initiative to foster networking and active discussion among PhD students.

Since 1959, SoIPa has organized a biennial congress that attracts participants from all over Italy, Europe and beyond. The conferences cover all aspects of general parasitology, including malaria, neglected tropical diseases, food and waterborne zoonoses, ectoparasites, endoparasites (protozoa and helminths), mycology, epidemiology of parasitic infections, parasites of companion animals, food-producing animals and wildlife, parasites of fish, molecular systematics and phylogeny of parasites and vectors, population genetics, genomics, transcriptomics, proteomics, immunology, antiparasitic drugs and drug development, education and policy.

The XXXII Italian Congress of Parasitology (hereafter ‘the SoIPa Congress’) was held in Naples from 27th June to 30th June 2022, gathering nearly 250 researchers and professionals, with the aim of exploring the new role that parasitologists can play in the future society, considering the period of rapid changes and transitions in the different fields of parasitology (Stothard *et al*., 2018) combined with social, economic, environmental, geopolitical, digital and technological transformations that currently govern global health.

**Figure 1.**
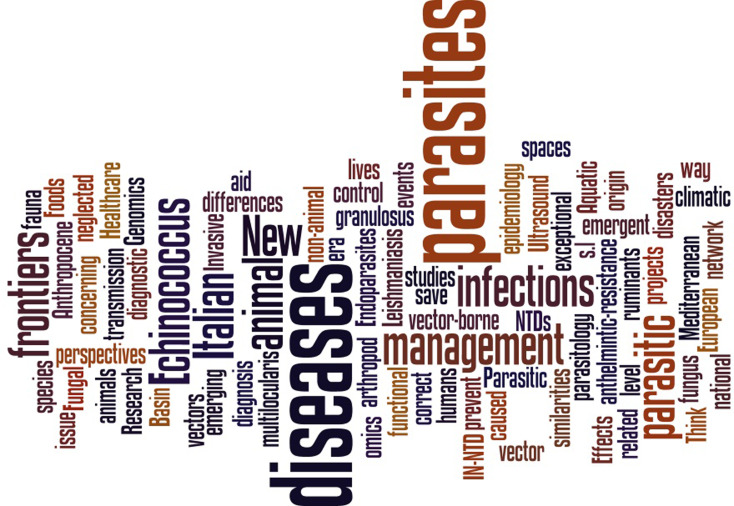
Word cloud based on the words (*n* = 143) used in the 14 symposia titles during the XXXII SoIPa Congress (prepared using Wordle^TM^ – https://www.wordle.net/).

The present Special Issue (SI) contains some selected outcomes of the emerging topics presented and discussed during the symposia organized within the Congress. Many senior members of SoIPa actively participated in the symposia organization, identifying the most innovative and ‘hot’ topics and involving junior parasitologists. The frequent use of the terms ‘new’ and ‘frontiers’ in the titles of the symposia, as shown in Fig. 1, once again underlines the transitional nature of the current times, highlighting the necessity also for parasitologists to be prepared for tackling the new challenges of a changing world.

## Symposia and contributions: a dynamic and multi-faceted scientific framework

The main aim of the SoIPa Congress was to facilitate reciprocal update of the members of the Society, creating the right place for presenting relevant outcomes of recent and ongoing research and projects. These occasions stimulate discussion and peer-review analysis of implemented methods and obtained results, building a continuing education process that ultimately fosters a collaborative environment and improves the scientific quality of the research outputs. The Congress featured 14 symposia, proposed by 24 senior scientists, members of the Society, through a bottom-up process. A call for organizing symposia on emerging and internationally relevant topics was launched several months before the Congress to encourage cross-cutting participation from different research groups and the inclusion of key international speakers. Overall, 81 researchers were invited to contribute to the symposia, 16 of which were among the internationally recognized experts, leading parasitology research teams in foreign institutions in Australia, Brazil, France, Germany, Norway, Portugal, Spain, Switzerland, the UK and the USA.

The contents of 6 symposia were selected to be transformed into review papers and included in this SI, taking into account the novelty of their approach, as described in the following section.

In parallel with the 14 symposia, a total of 167 abstracts were submitted, of which 94 were presented as oral communications and 73 as posters. The contributions presented during the SoIPa Congress were divided in different topics, with the most attractive topic being ‘Vectors and Vector-Borne Diseases’ (*n* = 43 abstracts), witnessing the increasing relevance of vectorial transmission of pathogens in a world undergoing an alarming climate change. The second most appealing topic in terms of number of contributions was ‘Zoonoses and One Health’ (*n* = 33), confirming the strong interaction between medical and veterinary parasitology. The other topics were identified based on the host (i.e. ‘Companion animal parasitic diseases’, ‘Farm animal parasitic diseases’, ‘Medical Tropical parasitic diseases’, ‘Parasitic diseases in aquatic animals’, ‘Parasitic diseases in wild and exotic animals’; overall *n* = 71) or focusing on specific aspects of parasitological research (i.e. ‘Antiparasitic drugs: efficacy and resistance’, ‘Innovative diagnosis of parasitic diseases’, overall *n* = 13). Furthermore, a session was devoted to ‘Mycotic diseases’ (*n* = 7). Special sessions (*n* = 4) for early career parasitologists (*n* = 29) were held as part of an award competition promoted by the Society.

Finally, the Congress was introduced by the opening ceremony with 2 keynote speakers, namely Maria Elena Bottazzi (Center for Vaccine Development, Baylor College of Medicine, Houston, TX, USA) and Jürg Utzinger (Swiss Tropical and Public Health Institute, University of Basel, Switzeland). The former gave an overview of the past, present and future of parasite vaccine development, accelerated by the Covid 19 experience. Prof. Utzinger presented key developments over the past 75 years with particular emphasis on diagnostics, spatially explicit risk profiling, treatment and integrated control and elimination of helminth infections. The Congress was also enriched by a key-note by John Russell Stothard (Liverpool School of Tropical Medicine, UK) on a recent investigation of the public health relevance of the parasitic fauna of semi-captive baboons hosted in a safari park in UK, which is also one of the selected manuscripts of this SI.

The proceedings of the Congress are freely available on the SoIPa website (https://www.soipa.it/2022/07/19/gli-atti-del-xxxii-congresso-soipa/).

## Selected outcomes from the symposia

### Italian network on NTDs (IN-NTD)

The list of 20 diseases and disease groups classified as Neglected Tropical Diseases (NTDs) by the World Health Organization (WHO) includes parasites (helminths, protozoa as well as ectoparasites), bacteria, viruses, fungi and toxins (https://www.who.int/teams/control-of-neglected-tropical-diseases/overview). While their impact is particularly heavy in Africa, Latin America and Asia, NTDs are nowadays seen with increasing frequency outside endemic regions, due to climate change, migrations and international travelling (Casulli, 2021). A symposium on NTDs was organized with the dual purpose of reviewing epidemiology of NTDs in Italy, with a focus on the parasitic infections currently or formerly endemic in Italy, and was also the occasion to present to a wider audience the ‘Italian network on NTDs’ (IN-NTD), a newly established coalition of institutions dedicated to fighting NTDs.

The manuscript by Casulli *et al*. (2023) provides a global prospective on WHO and NTDs and an up-to-date overview of the most relevant parasitic endemic or imported NTDs in Italy (including soil-transmitted helminths, schistosomiasis, strongyloidiasis, cysticercosis, cystic echinococcosis, Chagas disease and leishmaniasis).

Current public health interventions to combat NTDs face major challenges that can only be overcome through networking at the national and international levels. As an example, WHO encourages its collaborating centres to work more closely together to ensure the translation of research-based evidence into policy, practice and public health advancements. Based on these assumptions, the IN-NTD was established as an alliance of scientific societies (including SoIPa), institutes, foundations, universities and non-profit organizations united to fight NTDs at the national and international levels. The aim of the network is to coordinate the work of many in order to achieve the necessary synergy and authority to propose and organize useful actions to combat NTDs in Italy, as well as in the countries where NTDs are endemic through various tasks such as advocacy, training, research, international public health cooperation and sustainable development goals (Casulli *et al*., 2023).

### Wildlife parasites and citizen science

The importance of parasites in changing ecosystems was discussed during the symposium dedicated to wildlife and health management from a European perspective. Particular emphasis was given to the zoonotic parasites of wildlife, the interface between wildlife and livestock and the need for innovative approaches to harmonize wildlife population monitoring and disease health surveillance that engage the public. To this end, the citizen science approach, i.e. the collaboration of citizens in science, has allowed large-scale and cost-effective research in the field of zoonotic parasites of wildlife in recent years (Doherty *et al*., 2021). Incorporating citizen science into wildlife health surveillance can be beneficial, especially when resources are limited and cost-effectiveness is paramount (Lawson *et al*., 2015). The importance and advantages of citizen science is underlined in the paper of this SI dealing with wildlife and their role in the circulation of arthropods and vector-borne agents of zoonotic relevance (Sgroi *et al*., 2023).

Innovative approaches to the study of parasites in exotic wildlife are also the main theme of another article in this SI, dealing with gastrointestinal parasites in captive olive baboons at Knowsley Safari Park in Merseyside, northwest England (Juhasz *et al*., 2023). Fecal samples of baboons were collected from vehicles and sleeping areas, and video analysis of baboon–vehicle interactions was conducted during the study. The results showed the presence of the soil transmitted helminth parasites *Trichuris trichura* (48.0%) and *Strongyloides fuelleborni* (13.7%). The implications of these parasitological findings are discussed in terms of animal, keeper and public health, pointing to the need for a One Health approach in the control of neglected parasitic diseases.

### Comparing approaches to parasitological issues

Two symposia are represented in this section. The comparison of different approaches to parasite control in different species and close relatives is emerging as a narrative thread in research lines in Italy and abroad. One symposium focused on comparing epidemiological aspects and strategies of parasite control in phylogenetically close species, such as horse and donkey, cattle and buffalo, cats and wild cats, humans and non-human primates. The comparative approach, if systematically applied on the basis of sound and evidence-based knowledge, helps in the identification of inappropriate approaches to 1 species (usually the less common or economically relevant) because of incautious knowledge transfer from the other species. The importance of these types of studies and topics is underlined in this SI through the example of horse and donkey parasitology (Buono *et al*., 2023). Although these 2 host species can be infected by a similar range of parasites, they have important differences in the physiological response (i.e. donkeys typically show fewer clinical signs than horses, when heavily infected) and in drug pharmacokinetics (i.e. donkeys may require higher concentrations or shorter dosing intervals than horses). As a consequence, different thresholds (e.g. in terms of egg output for strongyles) and different drug dosages should be applied in the 2 animal species, calling for specific studies and knowledge for each of the 2 hosts.

From another perspective, a systematic and transversal comparison of parasite control practices promoted by different research groups in different geographical contexts can support the identification of strengths and weaknesses of each approach, leading to the fine-tuning of best practices and common guidelines. Comparison of control strategies for the most prevalent and economically important parasites of ruminants (i.e. gastrointestinal helminths) was the objective of another symposium held during the congress. The usefulness of comparing and discussing control practices has already been demonstrated at the European level through different collaborative projects (Charlier *et al*., 2022), however this exercise is still lacking at the national level. The Italian perspective on the control of gastrointestinal helminth infections was deeply analysed during the symposium and subsequently reported in this SI (Maurizio *et al*., 2023). The work includes a nationwide epidemiological update on parasite prevalence and on the possible emergence of anthelmintic resistance. The outcomes of this review represent an interesting point of view for other Mediterranean countries, where similar management systems and overlapping climatic conditions are found.

In both papers, the in-depth analysis of current practices and common knowledge leads to the identification of misbeliefs and inappropriate practices, and contributes to build the right approach on an evidence-based knowledge. These 2 manuscripts are highly relevant for the real world of the veterinary profession and farm advisors, aiming to change the attitude of farmers and veterinary practitioners, towards a species-specific approach to helminth control, integrating the intelligent use of chemical drugs with the adoption of alternative practices.

### Unusual perspectives on the role of parasites

Parasites constitute a substantial proportion of the biotic component in all ecosystems, in terms of both number of species and biomass. Notwithstanding their important ecological role, parasites are rarely considered in environmental conservation programs and their risk of extinction is often neglected (Lymbery and Smit, 2023). Two symposia of this Congress shared this unusual perspective on parasites, investigating how extreme events or long-term changes in the ecosystem, both natural and anthropogenic in origin, can impact parasite population dynamics.

The first symposium focused on the marine ecosystem and more specifically on the Mediterranean Sea, which is significantly affected by anthropogenic stressors, such as fish resource overexploitation, maritime transport and land-based pollutants/contaminants (Mandić and Piraino, 2023), as well as by the fast increase of water temperature and climate change. The Mediterranean Sea represents a hotspot of marine biodiversity, with complex networks and trophic chains in which different animal species, encompassing a multitude of taxa and phyla, found themselves interlaced and inter-dependant. Trophically transmitted helminths are associated to these trophic chains, having organisms in higher trophic-levels as definitive hosts. These parasites are indirectly impacted by ecosystem perturbations that may affect their intermediate, paratenic or definitive hosts, since they require stable food webs to complete successfully their life cycles. As a consequence, these helminths may serve as sentinels of the status of Mediterranean ecosystem, and the study of their host–parasite relationships can provide measurable indicators for monitoring the magnitude of anthropogenic impact on the environment. Specific examples are provided in the review by Palomba *et al*. (2023) of this SI, which developed the discussion held during the Symposium.

The second symposium dealing with this topic investigated how extreme weather events such as floodings, droughts and fires impact on parasite diffusion and transmission risks. These catastrophic events are increasing in frequency and intensity worldwide due to global warming, however the Mediterranean basin, including Italy, is considered a hot spot of climate change consequences (MedECC, 2020). The paper by Poglayen *et al*. (2023) summarises the main findings of the impact of 6 catastrophic events that occurred in Italy between 1976 and 2021, concluding with a few suggestions based on the lessons learned from the management of the above-mentioned events. Floodings can obviously facilitate the wide diffusion of environmental stages of waterborne protozoa (e.g. cysts of *Giardia intestinalis*, oocysts of *Cryptosporidium* spp. and *Toxoplasma gondii*) and other water- and soil-borne helminthic parasites (e.g. *Fasciola hepatica*) leading to possible epidemics, as evidenced by outbreaks recorded in Sardinia. By contrast, fires and droughts normally lead to a reduction of propagation forms (e.g. oocysts, larvae) and intermediate hosts (oribatid mites, xerophilous gastropod mollusks) and vectors (ixodid tick environmental stages), suggesting a beneficial (or cost) effect for the hosts. However, fires cause soil hydrophobicity increasing the risk of subsequent floodings through heavy rainfalls, and both events may influence movements, distribution and management practices for animals kept extensively (due to emergency reasons), leading to unexpected changes in parasite burdens or to the re-emergence and spread of parasitic diseases. Regular monitoring of the parasitological status of these animals can prevent clinical outcomes. The authors are therefore promoting a broader discussion about the so-called ‘disaster parasitology’, enlarging to a global scale the need for experience sharing, in order to guide priorities in the prevention and control of the parasitological implications of these catastrophic events. Finally, the use of geospatial technologies (Rinaldi and Cringoli, 2014), associated with new electronic devices, such as drones and data loggers, is reported as a useful tool in the assessment of climate change and extreme event impacts on parasite transmission.

## Conclusions/future directions for parasitological studies

The selected outcomes from the XXXII SoIPa Congress show how parasitological research plays a crucial role in addressing the challenges related to climate change, societal changes and digitalization. Also in the parasitology field, researchers and policy makers should work together to promote dialogue on science-informed decisions to support the so-called ‘twin transition’ (green and digital transitions).

Furthermore, education and multidisciplinarity constitute the triggers of medical and veterinary parasitological research. That is why ‘Education and future in parasitology’ and ‘Current Parasitology: Multi-disciplinarity’ are the themes of the XXXIII SoIPa Congress (Padova, Italy, June 2024) and of the 14th European Multicolloquium of Parasitology (EMOP, Wrocław, Poland, August 2024), respectively. In conclusion, SoIPa supports collaboration with other national and international scientific societies and public health institutions and agencies to promote parasitology in education, research, and public engagement.

## Data Availability

Data supporting the findings of this manuscript are available in the cited bibliography.
